# NRBF2-mediated autophagy contributes to metabolite replenishment and radioresistance in glioblastoma

**DOI:** 10.1038/s12276-022-00873-2

**Published:** 2022-11-04

**Authors:** Jeongha Kim, Hyunkoo Kang, Beomseok Son, Min-Jung Kim, JiHoon Kang, Kang Hyun Park, Jaewan Jeon, Sunmi Jo, Hae Yu Kim, HyeSook Youn, BuHyun Youn

**Affiliations:** 1 Department of Integrated Biological Science, Pusan National University, Busan, 46241 Republic of Korea; 2 siRNAgen Therapeutics, Daejeon, 34302 Republic of Korea; 3 Nuclear Science Research Institute, Pusan National University, Busan, 46241 Republic of Korea; 4 Department of Hematology and Medical Oncology, Winship Cancer Institute, Emory University School of Medicine, Atlanta, GA USA; 5 Department of Chemistry, Pusan National University, Busan, 46241 Republic of Korea; 6 SoulDot Co., Ltd, Pusan National University, Busan, 46241 Republic of Korea; 7 Department of Radiation Oncology, Haeundae Paik Hospital, Inje University School of Medicine, Busan, 48108 Republic of Korea; 8 Department of Neurosurgery, Haeundae Paik Hospital, Inje University College of Medicine, Busan, 48108 Republic of Korea; 9 Department of Integrative Bioscience and Biotechnology, Sejong University, Seoul, Republic of Korea; 10 Department of Biological Sciences, Pusan National University, Busan, Republic of Korea; 11 Present Address: Department of Naval Architecture and Ocean Engineering, Pusan National University, Busan, 46241 Republic of Korea

**Keywords:** Cancer metabolism, Prognostic markers

## Abstract

Overcoming therapeutic resistance in glioblastoma (GBM) is an essential strategy for improving cancer therapy. However, cancer cells possess various evasion mechanisms, such as metabolic reprogramming, which promote cell survival and limit therapy. The diverse metabolic fuel sources that are produced by autophagy provide tumors with metabolic plasticity and are known to induce drug or radioresistance in GBM. This study determined that autophagy, a common representative cell homeostasis mechanism, was upregulated upon treatment of GBM cells with ionizing radiation (IR). Nuclear receptor binding factor 2 (NRBF2)—a positive regulator of the autophagy initiation step—was found to be upregulated in a GBM orthotopic xenograft mouse model. Furthermore, ATP production and the oxygen consumption rate (OCR) increased upon activation of NRBF2-mediated autophagy. It was also discovered that changes in metabolic state were induced by alterations in metabolite levels caused by autophagy, thereby causing radioresistance. In addition, we found that lidoflazine—a vasodilator agent discovered through drug repositioning—significantly suppressed IR-induced migration, invasion, and proliferation by inhibiting NRBF2, resulting in a reduction in autophagic flux in both in vitro models and in vivo orthotopic xenograft mouse models. In summary, we propose that the upregulation of NRBF2 levels reprograms the metabolic state of GBM cells by activating autophagy, thus establishing NRBF2 as a potential therapeutic target for regulating radioresistance of GBM during radiotherapy.

## Cancer: Managing radiotherapy resistance in brain tumors

A drug that blocks autophagy, the process by which cells clear out damaged components, may help to overcome radiotherapy resistance in glioblastoma (GBM). GBM is an aggressive type of brain tumor, and radiotherapy is the treatment of choice, but tumors often develop resistance. Although resistance has been linked to autophagy, the mechanism is poorly understood. BuHyun Youn at Pusan National University, Busan, and HyeSook Youn at Sejong University in Seoul, South Korea, and coworkers found that after radiotherapy, cancer cells showed higher levels of NRBF2, a protein that triggers autophagy, and were more aggressive. Further investigation showed that cancer cells could reprogram their metabolism to use fuel sources made available by autophagy. Mice treated with lidoflazine, an NRBF2 inhibitor, showed improved survival and smaller tumors. Lidoflazine shows promise for managing radiotherapy resistance in GBM.

## Introduction

Glioblastoma (GBM) is a refractory cancer and the most severe form of primary tumor that occurs in the brain. Current treatment options for this disease include surgery, radiotherapy, and chemotherapy^1^. Depending on the characteristics of the brain tissue, there are instances when the tumor mass cannot be completely resected during surgery. Therefore, radiotherapy is the primary treatment technique for GBM. However, malignant cancers, including GBM, have been reported to be resistant to radiotherapy, leading to reduced treatment efficacy^2–4^. To date, the exact mechanism underlying this radioresistance has not been fully elucidated, and the development of effective radiosensitizers remains insufficient. Therefore, there is a growing need to identify the routes by which radioresistance is acquired, which is common in various types of GBM, and consequently the development of effective radiosensitizers that can control this phenomenon.

Cancer is closely associated with metabolic disorders^5,6^. Metabolic reprogramming is considered one of the hallmarks of cancer^7,8^. Aberrant activation of oncogenes and tumor-related signaling pathways can induce metabolic reprogramming^9,10^. Furthermore, the inactivation of tumor suppressor genes is an important factor underlying tumor metabolic changes, which can promote the development and progression of cancer^11,12^. Radiotherapy has been observed to primarily depend on glucose metabolism^13^, and mitochondrial metabolic alterations may also be involved in this process. Various mechanisms have been proposed as the cause of metabolic reprogramming in cancer cells; however, contemporary studies have focused specifically on autophagy. Several studies have highlighted the diverse substrates that can be degraded via autophagy, possessing the potential to fuel nearly all aspects of central carbon metabolism^14–16^. A comprehensive analysis of the metabolic pathways of patients with cancer who underwent radiotherapy revealed an increased expression of genes that regulate mitochondrial functions, autophagy, and lysosomal degradation activities, as well as a strong reliance on mitochondrial respiration, accompanied by a diminished dependence on the Warburg effect^17^. Although the significance of autophagy in inducing metabolic change has already been elucidated, the exact mechanism underlying the metabolic changes caused by autophagy that leads to radiation resistance remains unknown.

Autophagy is a degradation and clearance process that is activated to maintain cellular homeostasis. Several studies have demonstrated that autophagy is increased in cancer cells with radioresistance, and it has been further reported that radioresistance is acquired by enhancing autophagy via radiotherapy^18^. Chaachouas et al. analyzed both radioresistant and radiosensitive breast cancer cells and revealed that radioresistant cells exhibit a strong post-irradiation induction of autophagy, which consequently serves as a protective and prosurvival mechanism in radioresistance^19^. Furthermore, Lomonaco et al.^20^ analyzed two different GBM specimens and revealed that CD133^+^ glioma stem cells (GSCs) exhibited a higher level of autophagy and increased radioresistance. Although radiation-induced autophagy mediates radioresistance in GBM, the specific molecular mechanisms of autophagy remain unclear. Moreover, several critical side effects have been reported for chloroquine (CQ), and improvements in radiotherapy have not been identified^21^. This further highlights the necessity for a more in-depth study on the relationship between radioresistance and autophagy — particularly, the specific molecular mechanisms underlying them — rather than an overview of autophagy as a large-scale process.

Nuclear receptor binding factor 2 (NRBF2) is a protein 34 kDa in size that is present in the cytoplasm, nucleus, and autophagosome and is likely involved in starvation-induced autophagy via its interaction with VPS34 complex I. The VPS34 complex, the core component of class III phosphatidylinositol-3 kinase (PI3K-III), binds to Atg14L to control the initiation steps of autophagy. It was found that NRBF2 binds to VPS34 and interacts with beclin-1 and ATG14L, which are key factors in autophagy. Moreover, NRBF2 is known to play a major role in the initiation of autophagy^22,23^. It was recently reported that NRBF2 regulates the chemoresistance of lung cancer, but its role in GBM has not been elucidated^24^. As such, NRBF2 is a poorly studied target, but considering the known functions of NRBF2, it holds the potential to be a contributing factor in the autophagy process for promoting cancer survival.

## Materials and methods

### Antibodies and reagents

Primary antibodies specific for LC3B were purchased from Cell Signaling Technology (Cell Signaling Technology, Beverly, MA, USA), and those specific for NRBF2, Ki67, GAPDH, and β-actin were purchased from Santa Cruz Biotechnology (Santa Cruz Biotechnology, Santa Cruz, CA, USA). Antibodies specific for VPS34 were purchased from Echelon Biosciences (Echelon Biosciences, Salt Lake City, UT, USA), and antibodies specific for ATG14L were purchased from MBL (MBL, Tokyo, Japan). Secondary antibodies specific for mouse IgG and rabbit IgG were purchased from Enzo Life Sciences (Enzo Life Sciences, Ann Arbor, MI, USA). Dulbecco’s modified Eagle’s medium (DMEM), RPMI-1640, Hanks’ balanced salt solution (HBSS), phosphate buffered saline (PBS), and fetal bovine serum (FBS) were acquired from WelGENE Inc. (WelGENE Inc., Daegu, Korea). Penicillin‒streptomycin was obtained from Thermo Fisher Scientific (Thermo Fisher Scientific, Cleveland, OH, USA). siRNA specific for human NRBF2 and control siRNA were purchased from Bioneer (Bioneer, Daejeon, Korea).

### Cell lines, cell culture, and irradiation

The U-87 MG and A172 cell lines were obtained from the Korea Cell Line Bank (KCLB, Seoul, Republic of Korea). The phenotypes of these cell lines were authenticated using KCLB. The cells were cultured in RPMI-1640 (Cat# LM011-01), RPMI no glucose (Cat# LM011-60), DMEM (Cat# LM001-05), or DMEM no glucose (Cat# LM001-79) supplemented with 10% FBS, 10,000 U/mL penicillin‒streptomycin (Cat# 15140-122), and 100 mg/mL streptomycin at 37 °C in 95% air and 5% CO_2_. Subsequently, the samples were irradiated with M-150WE (Softex, Tokyo, Japan).

### Patient-derived GBM cell line generation

Patient-derived GSC11 GBM stem cells (GSCs) were provided by Dr. Frederick F. Lang (Department of Neurosurgery, The University of Texas, M.D. Anderson Cancer Center, Houston, TX, USA). In addition, patient-derived BCL20-HP02 and BCL21-HP03 GSCs were obtained from patients undergoing resection in accordance with a protocol approved by the Haeundae Paik Hospital (Inje University, Busan, Republic of Korea). BCL20-HP02 GSCs were derived from a 38-year-old male patient with GBM, and BCL21-HP03 GSCs were obtained from a 67-year-old male patient with GBM. Detailed patient information is summarized in Supplementary information, Supplementary Table 1. After resection, ~200–500 mg of tumor samples were collected into a tube containing DMEM/F-12 supplemented with B27. The tumor specimen was subsequently washed with 5 ml of HBSS to remove blood and debris. Following this, the tumor was dissected into small fragments and further minced into ~1 mm^3^ fragments using a sterile scalpel blade. To dissociate the GBM tumor tissue, the minced tumor fragments were treated with collagenase D (1 mg/ml) and DNase I (0.1 mg/ml) in HBSS and incubated at 37 °C for 30–90 min with gentle mixing. Finally, the solution was passed through a 70-μm sterile mesh filter to remove any large, undigested tumor pieces. To culture cells as tumor spheres, patient-derived GSCs were cultured in DMEM/F-12 supplemented with B27, EGF (20 ng/ml), bFGF (20 ng/ml), and penicillin‒streptomycin (10,000 U/ml) at 37 °C in a humidified atmosphere containing 95% air and 5% CO_2_.

### Animal care protocol and establishment of an orthotopic xenograft mouse model

Six-week-old male BALB/c athymic nude mice (Orient Bio, Seongnam, Republic of Korea) were used to generate a xenograft mouse model. The protocols followed were approved by the Institutional Animal Care and Use Committee of Pusan National University (Busan, Republic of Korea), and experiments were performed in accordance with the provisions of the National Institutes of Health’s Guide for the Care and Use of Laboratory Animals. The mice were maintained in animal care facilities in a temperature-regulated room (23 ± 1 °C) with a 12 h:12 h light-dark cycle. All animals were fed water and standard mouse chow ad libitum. Analyses were performed in a single-blind fashion, whereby the investigator was unaware of which treatment was administered to the mice. U-87 MG or ΔNRBF2 U-87 MG cells were harvested via trypsinization and suspended in serum-free media at a density of ~1 × 10^5^ cells/μL. Thereafter, ~1 × 10^6^ cells were stereotactically injected into the brains of the mice. Fourteen days after the injection date, the brains of the mice were irradiated with 2 Gy daily for 5 days using an X-ray generator True Beam STx. At the end of the treatment period, the animals were euthanized, and brain samples were harvested. The survival of mice is presented as Kaplan–Meier survival curves. *P* values were calculated using the Mantel–Cox test.

### Invasion and migration assay

The effects of NRBF2 knockdown and irradiation on cell invasion and migration capacity were investigated by transwell cell invasion/migration assays, as previously described^25^. Cells (2 × 10^5^ in serum-free medium containing 1 mM glucose) were seeded into the upper chambers of a 24-well Transwell chamber (Corning, Corning, NY, USA) fitted with a 5 μm pore-size insert and treated with NRBF2 siRNA and/or radiation for 24 h. Next, the medium in the lower chamber was changed to medium containing 10% FBS. After 12 h, the upper membrane surface was wiped with a cotton swab to remove cells that had not migrated into the lower side of the membrane. Then, the upper chambers were fixed with 70% EtOH, stained with 0.05% crystal violet, and photographed with an AXIO microscope from Carl Zeiss (Carl Zeiss, Oberkochen, Germany).

### Three-dimensional culture

One hundred percent growth factor-reduced Matrigel (BD Biosciences, Franklin Lakes, USA) was thawed overnight at 4 °C and thoroughly mixed using prechilled pipette tips before use. Cells were then cultured on eight-well chambered glass slides (Ibidi USA, Fitchburg, WI, USA) with Matrigel. The glass slides were incubated at 37 °C for 1 h, after which the cells were harvested, counted, and resuspended as a single-cell suspension of 25,000 cells/mL in the media. Next, 200 μL of the cell suspension was mixed with 200 μL of media containing 4% Matrigel and dispensed into each well of the glass slides. The cells were then incubated and allowed to attach at 37 °C in a 5% CO_2_ atmosphere for 3 days. For the immunofluorescence (IF) staining studies, the cells and acini were fixed with 2% paraformaldehyde for 20 min and then permeabilized in 0.5% Triton X-100 for 10 min. The cells and acini were subsequently washed three times with PBS, after which they were blocked in IF buffer (PBS with 0.1% BSA, 0.2% Triton X-100, and 0.05% Tween 20) containing 10% FBS at 37 °C for 30 min. Next, the samples were stained with primary anti-α-tubulin antibody overnight at 4 °C and then washed three times with IF buffer. Following incubation with DyLight 488-conjugated secondary antibodies (Thermo Fisher Scientific), the slides were mounted with Fluoroshield Mounting Medium with DAPI (Abcam, Cambridge, UK). Fluorescent images were visualized with an Olympus IX71 fluorescence microscope (Olympus Optical Co. Ltd.).

### Bioluminescence imaging of tumor xenograft growth

Bioluminescence imaging was conducted to monitor in vivo tumor growth using VISQUE Invivo Smart LF (Vieworks, Republic of Korea). Tumor-bearing mice were injected with 300 mg/kg of VivoGloTM Luciferin, In Vivo Grade (Cat# P1043) before isoflurane anesthesia. Radiance was measured 10 min after substrate injection.

### Hematoxylin and eosin staining and immunohistochemistry

Hematoxylin and eosin (H&E) staining and immunohistochemistry (IHC) analyses were performed as previously described^26^. Liver, lung, spleen, kidney, and brain samples were collected from the euthanized mice. The samples were then fixed in formalin, dehydrated, and embedded in paraffin. Subsequently, 4-mm-sized sections were cut and used for H&E or IHC analyses, following standard procedures. The stained sections were observed under an Axio Lab. A1 microscope (Zeiss, Germany).

### qRT‒PCR

Total RNA was extracted from GBM cell lines and orthotopic xenograft mouse brains using the TRIsure^TM^ RNA Cell Miniprep System (Cat#BIO-38033, Bioline, London, UK), and cDNA was synthesized using M-MLV Reverse Transcriptase (Cat#M1705, Promega). mRNA expression was measured by real-time PCR using the SensiFAST™ SYBR® Hi-ROX Kit (Cat#BIO-92020, Bioline, London, UK). Primer sequences are listed in Supplementary information, Supplementary Table 2.

### Generation of the NRBF2 knockout cell line

The GeneArt CRISPR Nuclease (OFP Reporter) Vector Kit (Cat# A21174, Thermo Fisher Scientific) was used for this project. Briefly, the designed single-stranded oligonucleotides were annealed and cloned into the CRISPR nuclease vector by using T4 DNA ligase. After transfection, the OFP-positive GBM cell lines were selected for FACS. gRNA of ATG7 and NRBF2 were obtained from previous data^27^ and http://guides.sanjanalab.org/#/. The establishment of stable NRBF2 KO cells was confirmed by western blotting.

### Measurement of oxygen consumption rate and extracellular acidification rate

The OCR and extracellular acidification rate (ECAR) were determined using a Seahorse XFp Analyzer (Agilent Technologies, Santa Clara, CA, USA). In brief, cells were plated on Seahorse XFp plates for 24 h at a concentration of ~6 × 10^3^ cells/well. Cells were subsequently washed and incubated with Seahorse XF Base Medium (supplemented with 5.5 mM glucose and 2 mM L-glutamine, pH 7.4) at 37 °C for 1 h in a non-CO_2_ incubator. For the OCR and ECAR assays, injection port A on the sensor cartridge was loaded with oligomycin (complex V inhibitor, final concentration 1 μM), port B was loaded with FCCP (final concentration 2 μM), and port C was loaded with rotenone/antimycin A (inhibitors of complex I and complex III, final concentration 0.5 μM each). Upon completion of the Seahorse XFp flux analysis, the cells were lysed to calculate the protein concentration. The results were normalized to the protein abundance in the corresponding wells. The data are representative of three biological replicates.

### Cell viability assay

For the cell viability assay, cells were seeded at 10,000 cells per well in 96-well plates 1 day before the addition of lidoflazine and/or IR for 48 h. Cell viability was determined using a CellTiter-Glo® Luminescent Viability Assay kit (Promega).

### Statistical analysis

All numerical data are presented as the mean ± standard error of the mean obtained from at least three independent experiments. For quantification, the data were analyzed using *t* tests or ANOVA. Prism 9 software (GraphPad Software, San Diego, CA, USA) was used for all statistical analyses. Statistical significance was set at *p* < 0.05. Detailed information about the materials and methods used in this study is provided in the supplementary information.

## Results

### An increase in NRBF2 expression was observed in glioblastoma after radiotherapy

**Fig. 1:**
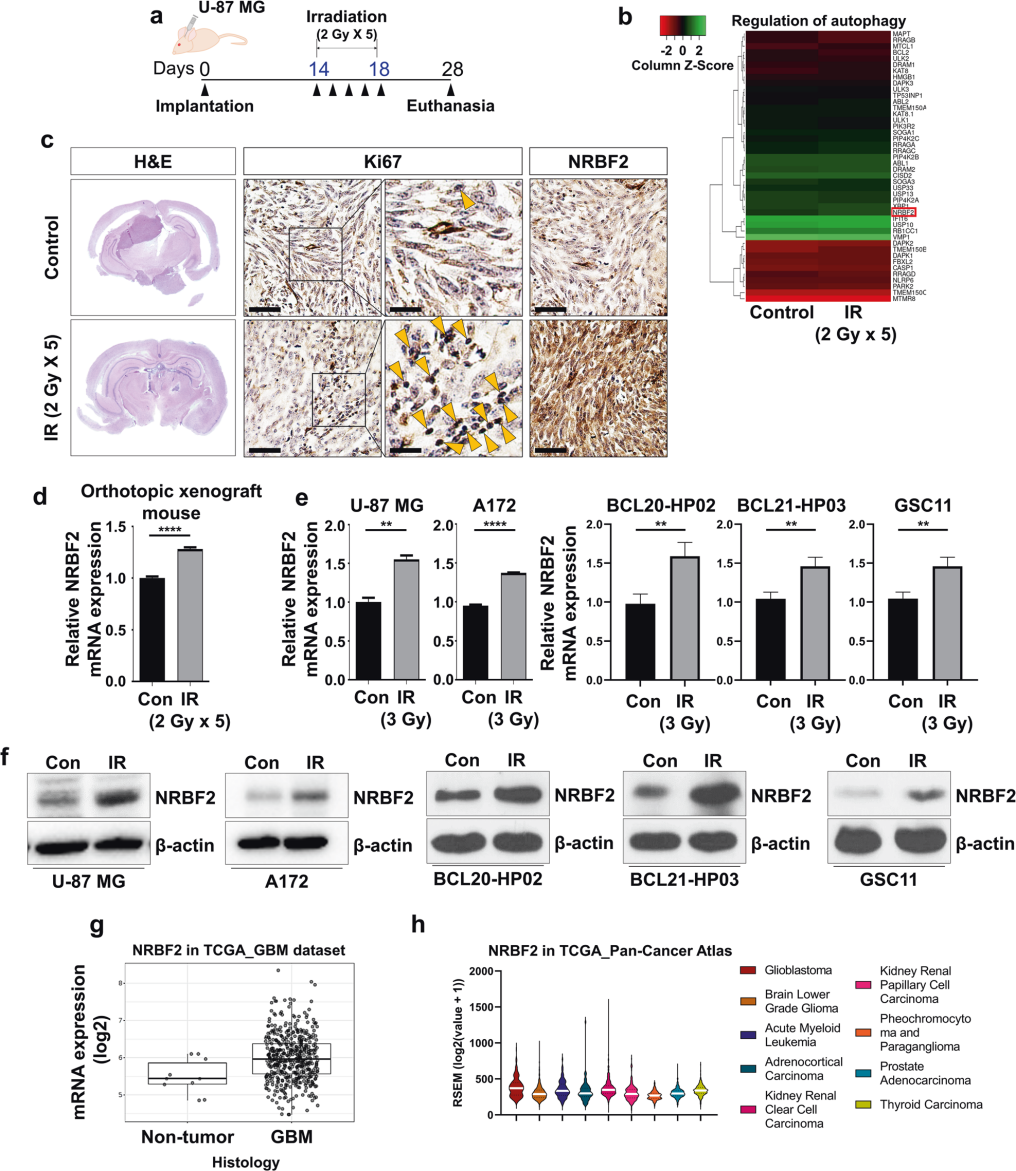
NRBF2 is upregulated after IR in glioblastoma. **a** Scheme of the U-87 Luc orthotopic xenograft mouse model and mouse irradiation schedule. **b** Microarray experiments were performed using cDNA samples obtained from orthotopic xenograft GBM tumor sections treated or not treated with 2Gy × 5 radiation, and microarray results were deposited in the Gene Expression Omnibus database (GEO: GSE117126). **c** Hematoxylin and eosin (H&E) staining (left panel) and immunohistochemistry (IHC) of Ki67 and NRBF2 (middle panel and right panel) of coronal sections from U-87 MG orthotopic xenograft mouse control or treated with IR (2Gy × 5). Scale bars, 10 μm. **d** Real-time qRT‒PCR analysis of the *nrbf2* mRNA levels in the GBM orthotopic xenograft mouse model. **e** Real-time qRT‒PCR analysis of the *nrbf2* mRNA levels in GBM cell lines. **f** Western blot analysis of control and IR-treated GBM cells shows IR-induced expression of NRBF2. **g** The gene expression profiles were collected from The Cancer Genome Atlas (TCGA) database available through Gliovis (PMID: 28031383). mRNA expression of *nrbf2* was measured in 10 nontumor tissues and 528 GBM tissues. **h** *Nrbf2* gene expression profiles were collected from The Cancer Genome Atlas (TCGA, Pan-Cancer Atlas) database (https://www.cbioportal.org/). mRNA expression of *nrbf2* was measured in nine different cancer types, including GBM. **p < 0.01, ****p < 0.0001 using an unpaired *t* test.

Many studies have reported that autophagy is involved in the acquisition of treatment resistance in various malignant tumors, particularly in pancreatic ductal adenocarcinoma (PDAC), and it has been further observed that inhibition of autophagy can be an effective combination therapy strategy. The correlation between therapy resistance and autophagy in GBM, another malignant tumor, has not been studied as well as in PDAC, but recent studies suggest that there is a close relationship between the two phenomena. Based on these outcomes, a study was conducted based on the hypothesis that the autophagy mechanism is crucial for acquiring radiation resistance in GBM^20,28,29^. Initially, microarray analysis was performed to identify key factors related to autophagy that were altered after radiation treatment in GBM orthotopic xenograft mice (GEO accession number: GSE117126) (Fig. 1a, b). We observed that the *nrbf2* gene was highly expressed in the irradiated group compared to the control group. (Fig. 1b). To further validate the microarray analysis results, U-87 MG cells were xenografted orthotopically, followed by radiotherapy after two weeks of implantation. After irradiation, the tumor exhibited a decrease in the IR (total 10 Gy) group, but it did not disappear completely. (Fig. 1c). Although the size of the tumor decreased in the IR group, it was confirmed that the expression of the proliferation markers Ki67 and NRBF2 was increased in the surviving tumor cells. (Fig. 1c, d). These results indicate that the tumor cells that survived and acquired radiation resistance after irradiation exhibited high tumorigenicity, with increased expression of NRBF2. In addition to the GBM orthotopic xenograft mouse model, the two most commonly used GBM cell lines (U-87 MG and A172) and three patient-derived primary GBM cell lines (BCL20-HP02, BCL21-HP03, and GSC11) were used to confirm the general alteration in the pattern of *nrbf2* expression after irradiation. Based on previous studies that considered the difference between cranial irradiation and cell irradiation, a dose of 3 Gy was used in in vitro experiments^30^. Interestingly, both the mRNA and protein expression of NRBF2 increased after 3 Gy irradiation in all cells (Fig. 1e, f). In addition to confirming alterations in NRBF2 through experiments based on GlioVis (http://gliovis.bioinfo.cnio.es, accessed 31 October 2016), a database related to brain tumor studies, it was confirmed that the expression of *nrbf2* was higher in the GBM group (Fig. 1g). Furthermore, when NRBF2 levels were evaluated in other carcinomas, the highest expression level was observed in GBM (Fig. 1h). These data demonstrated that alteration of NRBF2 expression after irradiation is not only common in mouse models as well as in vitro but also has clinical relevance, suggesting the possibility that NRBF2 may play a significant role in radioresistance and malignancy of GBM.

### Regulation of NRBF2 is necessary for GBM cell progression

**Fig. 2:**
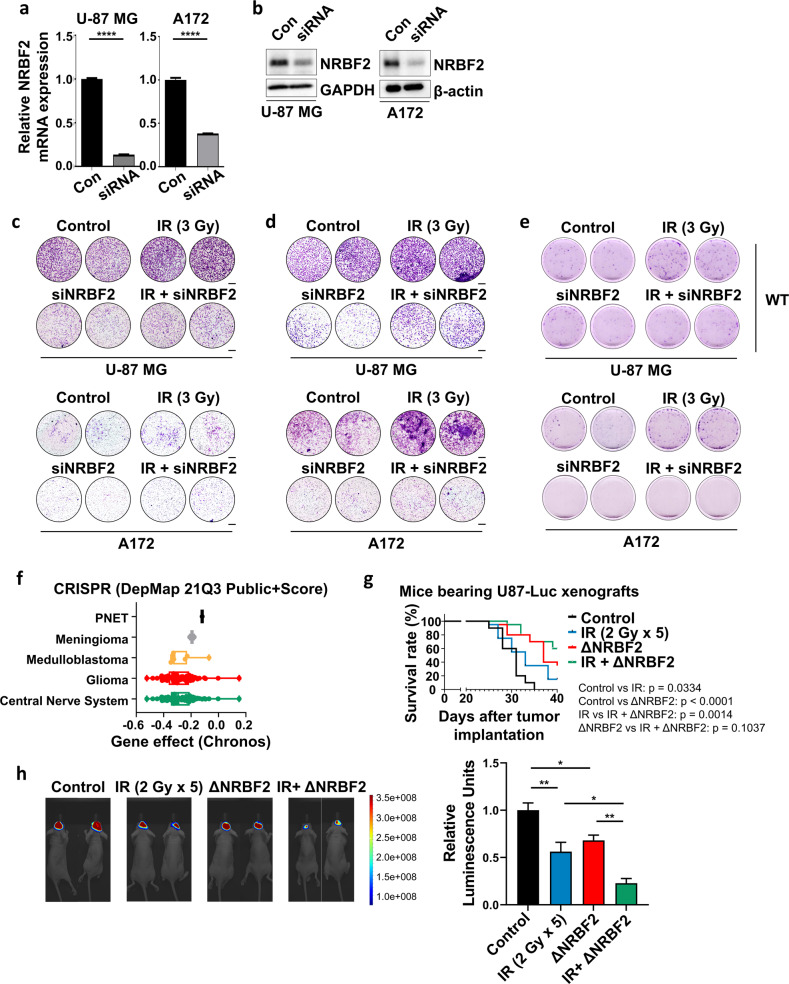
NRBF2 regulates cell aggressiveness, proliferation, and tumor growth. **a** Real-time qRT‒PCR analysis of *nrbf2* mRNA levels in U-87 MG and A172 cells after *nrbf2* knockdown. **b** NRBF2 protein levels after NRBF2 siRNA transfection confirmed by western blot. **c**, **d** Representative images of the transwell migration assay (**c**) and invasion assay (**d**) of GBM cell lines cultured in low glucose media containing 1 mM glucose with NRBF2 or IR (3 Gy) treatment siRNA or both. Scale bars, 200 µm. **e** Clonogenic assay after NRBF2 knockdown, IR (3 Gy) or both in GBM cells cultured in low glucose media containing 1 mM glucose. Representative images of single-cell clone proliferation stained with crystal violet. **f** DepMap (https://depmap.org/portal/) analysis of the expression of NRBF2 in CNS, glioma, medulloblastoma, and meningioma. **g** Survival graph of U-87 Luc WT or NRBF2 knockout orthotopic xenograft mouse model with radiation (2Gy × 5). Statistical analysis was performed with the log-rank (Mantel‒Cox) test. **h** Luminescence images (left) and relative luminescence units (right) of the indicated U87-Luc orthotopic xenografts.

To investigate whether NRBF2 regulates the progression of GBM, several cellular assays were performed. Before proceeding with the assays, siRNA was used to knockdown NRBF2 expression. qRT‒PCR and western blotting were used to verify the efficacy of NRBF2 siRNA by measuring RNA and protein levels, respectively (Fig. 2a, b). Furthermore, to investigate tumor progression and metastatic features after irradiation or knockdown of NRBF2^31,32^, migration and invasion assays were performed using U-87 MG and A172 GBM cell lines cultured in low glucose media containing 1 mM glucose. Following irradiation, cell migration and invasion were observed to be increased compared to those in the control group. However, these results were reversed when NRBF2 was knocked down. Treatment with siRNA and ionizing radiation attenuated cell migration and invasion (Fig. 2c, d).

In addition to these phenomena, cell proliferation also affects the aggressiveness of cancer, which is linked directly to poor prognosis and therapeutic resistance^33–35^. To investigate cell proliferation following the irradiation and knockdown experiments, clonogenic assays were performed in four groups: control, IR, siNRBF2, and both IR- and siNRBF2-treated groups. Interestingly, in normal glucose media, the proliferation rate decreased in the following order: control, IR, siNRBF2, and both IR- and siNRBF2-treated groups (Supplementary Fig. 1a). However, in low glucose media, the growth rate of the irradiation group was higher than that of the control group (Fig. 2e). NRBF2 is expected to be involved in autophagy, and since autophagy is activated at a higher rate in a low glucose environment, cell proliferation changes under the same conditions were studied—i.e., when NRBF2 is continuously maintained in a high environment.

To verify this in human cancers, we investigated the role of NRBF2 in vivo using the DepMap database. DepMap (https://depmap.org/portal/) analysis implied that NRBF2 is a key regulator of cell survival (Fig. 2f), which further validated our results. Using the NRBF2 knockout U-87MG cell line (Supplementary Fig. 1b, c), it was found that the ΔNRBF2 U-87MG xenograft mouse exhibited a higher survival rate than the U-87MG cell line. In addition, IR promoted the survival of the ΔNRBF2 U-87MG xenograft mice (Fig. 2g). As with the survival rate of the mouse model as well as in vitro experiments, tumor growth was the lowest in the group with the irradiated ΔNRBF2 U-87MG xenograft mouse model (Fig. 2h). These data demonstrate that NRBF2 plays a crucial role in regulating tumor progression and proliferation, as well as protecting tumor cells from radiation.

### NRBF2 binds the VPS34 complex and is involved in the autophagy initiation step

**Fig. 3:**
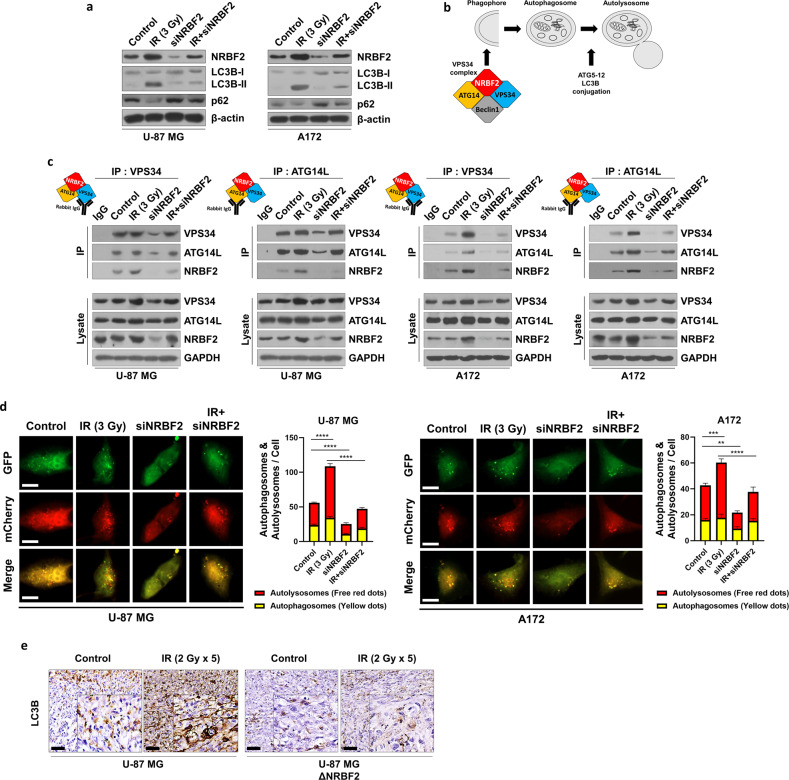
NRBF2 regulates autophagy through the VPS34 complex. **a** Autophagy-related protein levels after treatment confirmed by western blot. **b** Schematic images of the autophagy initiation step involved in NRBF2. **c** Coimmunoprecipitation of NRBF2 and the VPS34 complex confirmed by western blot in GBM cell lines. **d** Autophagic flux analysis using the mCherry-EGFP reporter. A172 and U-87 MG cells transfected with mCherry-GFP-LC3B were treated with IR (3 Gy), siNRBF2 or both. The mean numbers of autophagosomes and autolysosomes are represented by yellow and red dots, respectively, in the merged images. The images were quantified using ImageJ (*n* = 5). Scale bars, 10 µm. **e** IHC of LC3B in coronal sections from U-87 WT or NRBF2 knockout orthotopic xenograft mouse models treated with radiation (2 Gy × 5). Scale bars, 10 μm. **p < 0.01, ***p < 0.001, ****p < 0.0001 using an unpaired *t* test.

NRBF2 is a key regulator of phagophore and autophagosome formation (Fig. 3b)^23,36–38^. Based on these findings, the impact of NRBF2-related autophagic flux was assessed by measuring the levels of LC3B-II and p62, which are markers that can confirm autophagic flux. Consequently, LC3B-II expression was increased in the high NRBF2 expression group, whereas knockdown of NRBF2 after IR demonstrated low autophagic flux (Fig. 3a). A coimmunoprecipitation assay was performed to determine whether the activation of autophagic flux proceeded via a known mechanism in the NRBF2-upregulated group. As expected, upregulation of NRBF2 enhanced the formation of the initiation complex, while complex formation was reduced by downregulation of NRBF2 after irradiation (Fig. 3c). After corroborating NRBF2-mediated autophagy, autophagic flux was measured using an LC3B reporter (mCherry-GFP-LC3B WT), which labels autophagosomes in yellow (mCherry and GFP) and autolysosomes in red (mCherry)^39^. Immunofluorescence analysis revealed that activation of NRBF2 caused yellow and red dots to appear, which indicated the formation of autophagosomes and autolysosomes, respectively, but these punctate patterns were reduced in the NRBF2 knockdown group (Fig. 3d). After verifying the in vitro experiment, an in vivo study was performed in a GBM orthotopic xenograft mouse model. In concurrence with the in vitro results, LC3B expression was higher in the irradiated group than in the NRBF2 knockout group (Fig. 3e). These results indicate that radiotherapy upregulates NRBF2-mediated autophagy in GBM cells.

### NRBF2-mediated autophagy maintains GBM metabolism

**Fig. 4:**
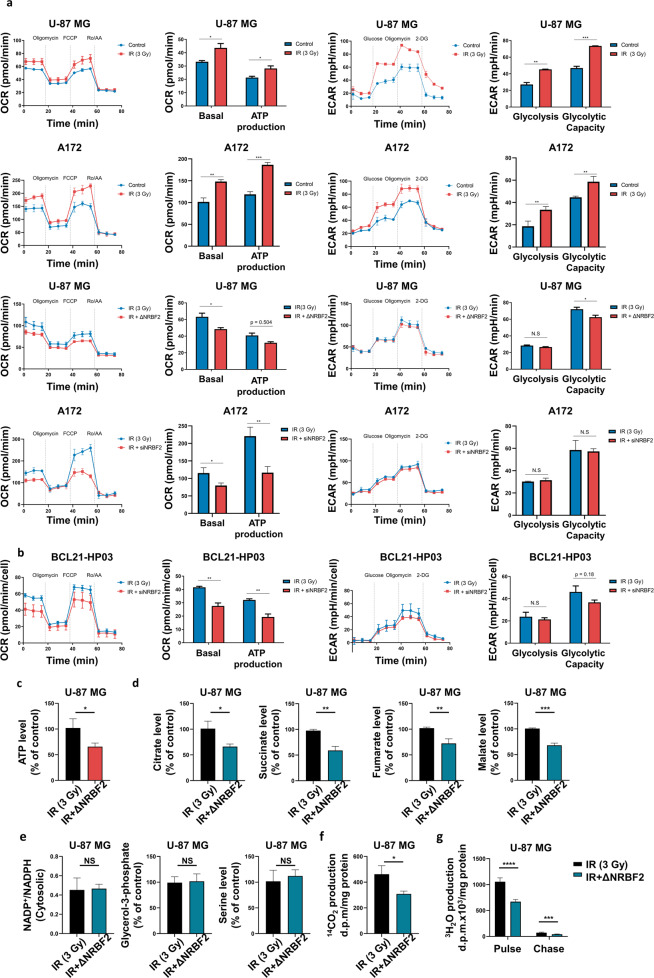
NRBF2-induced autophagy provides a metabolic advantage to GBM cells. **a** Oxygen consumption rates (OCR) and extracellular acidification rates (ECAR) were measured by a Seahorse XFp Analyzer in U-87 MG and A172 cells with or without IR (3 Gy), IR-treated U-87 MG cells with or without NRBF2 knockout, and IR-treated A172 cells with or without NRBF2 knockdown. **b** OCR and ECAR were measured in IR-treated patient-derived GBM cells with or without NRBF2 knockdown. **c**–**g** Each bar graph shows the metabolite levels in WT and NRBF2 knockout U-87 MG cells. **c** ATP levels were determined in WT and NRBF2 knockout U-87 MG cells after irradiation (3 Gy). **d** TCA cycle metabolites such as citrate, succinate, fumarate, and malate were detected. **e** Metabolites of glycolysis-related pathways were determined by measuring the NADP^+^/NADPH ratio, glycerol-3-phosphate, and serine levels. **f** WT and NRBF2 knockout U-87 MG cells were incubated with [1-^14^C] oleic acid, and ^14^CO_2_ production from the complete b-oxidation of [1-^14^C] oleic acid was analyzed. **g** After incubation with [9,10-^3^H] oleic acid (pulse) followed by incubation in DMEM (chase), media equivalent to 500 μg of cell protein were analyzed for radioactivity in the form of ^3^H_2_O. *p < 0.05, **p < 0.01, ***p < 0.001, ****p < 0.0001 using an unpaired *t* test.

Autophagy is essential for maintaining cellular homeostasis, including metabolism. This mechanism can be modulated under normal conditions or pathological processes, such as cancer. Recent studies have suggested that autophagy can provide diverse metabolic fuel sources for cancer cell maintenance and facilitate metabolic plasticity in cancer^14,15,40–43^. Therefore, the present study hypothesized that NRBF2-mediated autophagy could alter metabolite status. First, the metabolic potentials were evaluated by measuring the OCR and ECAR. Both OCR and ECAR levels were increased by IR, whereas only OCR levels were significantly decreased by NRBF2 knockout or knockdown in IR-treated GBM cells (Fig. 4a). Although radiation treatment elevated NRBF2 expression in GBM (Fig. 1), there was another reason for the increase in ECAR, apart from boosting NRBF2. Next, OCR and ECAR levels were confirmed in patient-derived GBM cells. As expected, ATP production was higher in the IR-alone group than in the NRBF2 knockdown group, and glycolysis showed no difference (Fig. 4b). These results indicate that NRBF2 affects TCA cycle metabolites rather than glycolysis metabolites.

For further validation, a metabolism assay was conducted based on Seahorse assay results. Since oxidative phosphorylation levels significantly decreased in ΔNRBF2 cells after IR (Fig. 3a), metabolite levels were measured in the NRBF2 WT and ΔNRBF2 groups. The levels of ATP and TCA cycle metabolites, such as citrate, succinate, fumarate, and malate, were decreased in ΔNRBF2 U-87 MG cells (Fig. 4c, d). However, no variation was observed in the metabolite levels of the glycolysis pathway (Fig. 4e). Additionally, oxidative phosphorylation occurs in the mitochondria. Therefore, ^3^H_2_O and ^14^CO_2_ release levels were measured under the assumption that mitochondrial β-oxidation affects OCR levels. The release of ^3^H_2_O and ^14^CO_2_ was monitored from [9,10-^3^H]oleic acid and [1-^14^C]oleic acid, respectively, and lower levels of ^3^H_2_O and ^14^CO_2_ were detected in the ΔNRBF2 U-87 MG cells, indicating that NRBF2 WT cells had high β-oxidation levels after radiation (Fig. 4f, g). Another GBM cell line, A172 NRBF2 knockdown cells, also exhibited a decrease in TCA cycle metabolites and ATP (Supplementary Fig. 2a–e). Notably, mitochondrial membrane potential was not affected by NRBF2 knockout, suggesting that the metabolic changes mediated by NRBF2 are not due to mitochondrial defects (Supplementary Fig. 2f). The Cancer Genome Atlas (TCGA) database was used to prove the relationship between NRBF2 and TCA cycle metabolites (Supplementary Fig. 2g). The correlation between the enzymes involved in the TCA cycle and NRBF2 was evaluated, and a positive correlation was confirmed. These results revealed that NRBF2 expression and oxidative phosphorylation were related to NRBF2-mediated autophagy activation.

### Lidoflazine enhanced radiosensitivity by inhibiting NRBF2-mediated autophagy

**Fig. 5:**
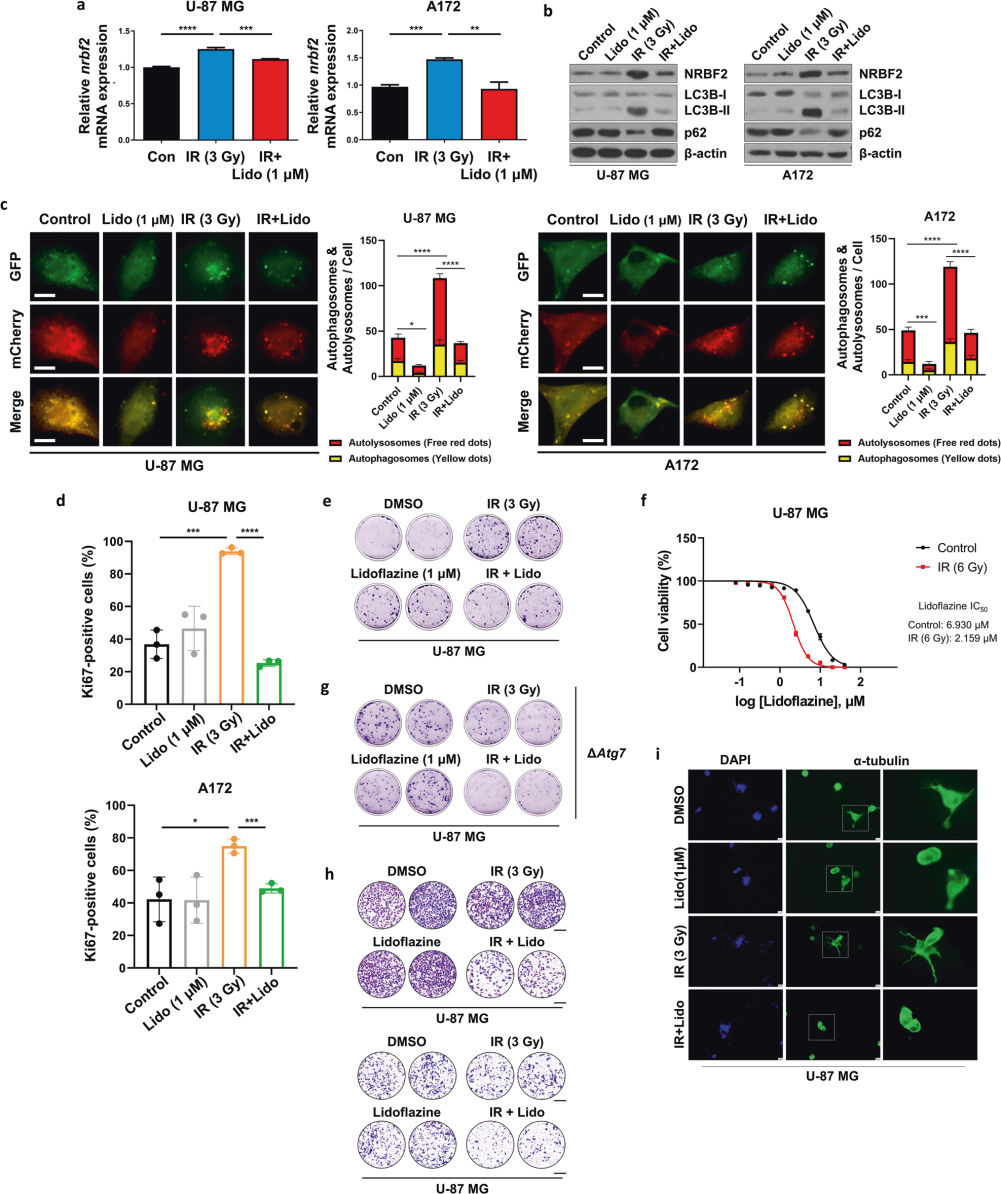
Lidoflazine increases the radiosensitivity of GBM cell lines. **a** qRT‒PCR analysis of *nrbf2* mRNA levels after irradiation and lidoflazine treatment in a GBM cell line. **b** Western blot analysis to evaluate autophagic flux after irradiation and treatment of lidoflazine in U-87 MG cells. **c** Autophagic flux analysis using the mCherry-EGFP reporter. U-87 MG and A172 cells transfected with mCherry-GFP-LC3B were treated with IR (3 Gy), lidoflazine (1 μM), or both for 24 h. The mean number of autophagosomes and autolysosomes are represented by yellow and red dots, respectively, in the merged images. The images were quantified using ImageJ (*n* = 5). Scale bars, 10 µm. **d** Percentage of GBM cells stained positively for the Ki67 proliferation marker with different treatments. Differences among means were statistically significant, according to a *t* test. **e** Clonogenic assay after IR (3 Gy), lidoflazine (1 μM), or both in U-87 MG cells cultured in low glucose media containing 1 mM glucose. Representative images of single-cell clone proliferation stained with crystal violet. **f** IC_50_ of lidoflazine with or without IR in U-87 MG cells was measured by CellTiter-Glo® Luminescent Cell Viability Assay. Cells were treated with increasing concentrations of lidoflazine and/or IR (3 Gy) for 48 h. **g** Clonogenic assay after IR (3 Gy), lidoflazine (1 μM), or both in ATG7 knockout U-87 MG cells cultured in low glucose media containing 1 mM glucose. Representative images of single-cell clone proliferation stained with crystal violet. **h** Transwell migration assay (upper) and invasion assay (lower) of U-87 MG cells cultured in low glucose media containing 1 mM glucose with treatment with lidoflazine (1 μM), IR (3 Gy), or both. **i** U-87 MG cells in a three-dimensional culture system were stained with α-tubulin (green) and DAPI (blue). Scale bars, 10 µm. *p < 0.05, ***p < 0.001, ****p < 0.0001 using an unpaired *t test*.

Since the increase in NRBF2 due to radiation promoted GBM aggressiveness, regulating NRBF2 activation is important for cancer treatment. To overcome this, a connectivity map (CMap) containing drug-specific gene expression profiles was utilized to search for drugs or compounds. Through CMap (https://clue.io/cmap), it was hypothesized that a drug called lidoflazine, which was originally used as a coronary vasodilator, would modulate NRBF2. To determine the appropriate drug concentration, an MTT assay was performed, whereby 1 µM was found to be a suitable concentration (data not shown). Lidoflazine treatment diminished NRBF2 mRNA and protein levels after radiation, but surprisingly, there was no effect of lidoflazine when administered alone (Fig. 5a, b).

NRBF2-mediated autophagy is one of the mechanisms by which GBM acquires radioresistance. Therefore, to confirm whether lidoflazine can decrease radioresistance by reducing autophagy, autophagic flux and cell proliferation levels were evaluated following treatment with lidoflazine. Using mCherry-GFP-LC3B, a very low autophagic flux was observed in the group treated with IR and lidoflazine (Fig. 5c). In addition, cell proliferation was confirmed using the proliferation marker ki67 and clonogenic assays, whereby low Ki67-positive levels and colony formation were confirmed in the group treated with lidoflazine and IR in the same context as autophagic flux (Fig. 5d, e and Supplementary Fig. 3a, b). We next tested the effect of IR on lidoflazine sensitivity using the CellTiter-Glo® Luminescent Cell Viability Assay (Fig. 5f). IR significantly improved lidoflazine sensitivity in U-87 MG cells, reducing the IC50 of lidoflazine from 6.930 μM to 2.159 μM. Interestingly, when ATG7, a key autophagosomal protein, was knocked out, cell proliferation decreased in the IR group, but the lidoflazine effect did not decrease (Fig. 5g and Supplementary Fig. 1d, e). This result indicated that autophagy is a key regulator of radioresistance and that lidoflazine could alleviate autophagic flux. Since NRBF2 was also involved in the aggressiveness of cancer cells (Fig. 2c), it was confirmed that lidoflazine could alleviate this malignant effect. A transwell assay was used to validate the migration and invasion of GBM cells, whereby a decrease in the same manner was observed in the group treated with radiation (Fig. 5h and Supplementary Fig. 3c). Subsequently, the effect of the lidoflazine was verified once again using a 3D culture system. 3D tumor cells have demonstrated numerous characteristics that vary from traditional 2D culture. 3D cell culture provides a useful platform for further identification of the biological characteristics of tumor cells, particularly in drug sensitivity, among other key aspects of translational medicine^44^. The reduced invasiveness was confirmed in the treated group, with the same results as obtained from the transwell assay performed in 2D cultures (Fig. 5i and Supplementary Fig. 3d). Overall, the group treated with lidoflazine alone showed no significant difference from the vehicle group, but a significant effect was observed in the group treated with radiation as well as lidoflazine. In summary, lidoflazine acts as a radiosensitizer by reducing NRBF2, which is increased by IR.

### Lidoflazine has a therapeutic effect on radioresistant cells

Based on the results that implicated the involvement of NRBF2 in radioresistance, radioresistant cells were constructed, and the effect of lidoflazine was assessed. First, a subcutaneous xenograft mouse model was established by introducing U-87 MG cells into BALB/c nude mice. When the tumor increased to a certain size, the mice were irradiated five times with a dose of 2 Gy each. Tumors were subsequently isolated from xenograft mice, and a primary culture was established. Thereafter, the cells were implanted subcutaneously into BALB/c nude mice and irradiated five times at the same dose. Finally, primary cells from the second subcutaneous tumor were introduced into orthotopic xenograft mice, and brain-specific radiation was administered to the established mice (Supplementary Fig. 4a). Clonogenic assay results suggested that the proliferation of U-87 MG-RR cells was much higher than that of U-87 MG cells, and it was confirmed that the same pattern was exhibited even after irradiation (Supplementary Fig. 4b). Since NRBF2 is a factor responsible for radioresistance, NRBF2 levels were evaluated in the constructed RR cells, and it was confirmed that both the mRNA and protein levels were increased in RR cells (Supplementary Fig. 4c, d).

**Fig. 6:**
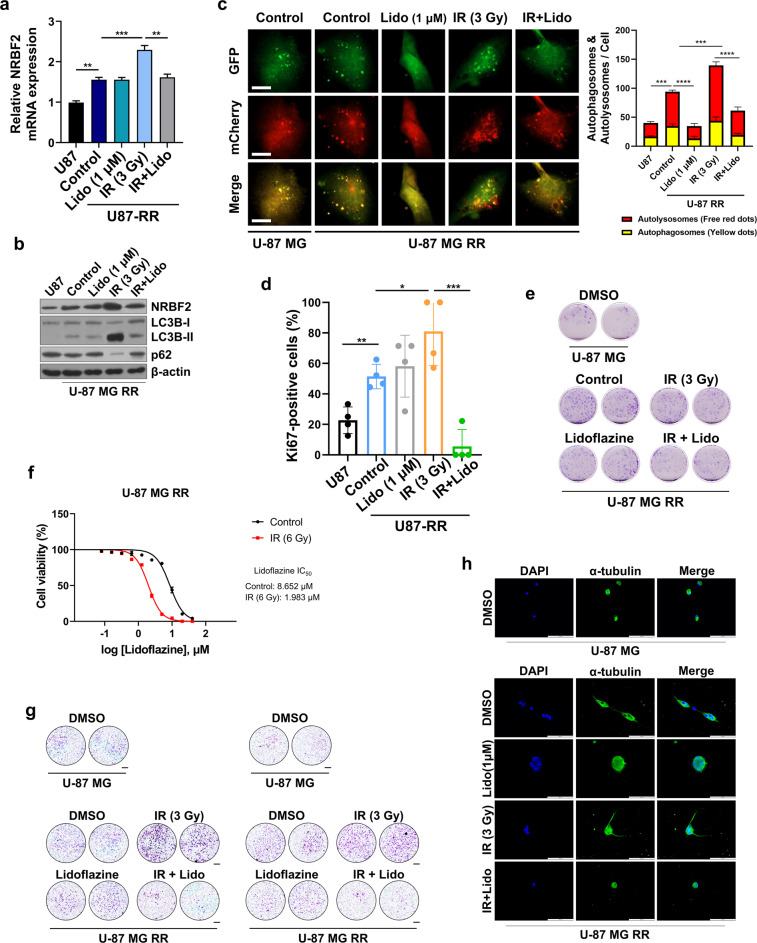
Cells with enhanced radioresistance acquire radiosensitivity by inhibiting NRBF2. **a** qRT‒PCR analysis of *nrbf2* mRNA levels after IR (3 Gy) or additional treatment with lidoflazine (1 µM) in RR cells or untreated U-87 MG cells. **b** Western blot analysis of NRBF2 protein expression levels after IR (3 Gy), lidoflazine (1 µM), or both in RR cells or untreated U-87 MG cells. **c** Autophagic flux analysis using the mCherry-EGFP reporter. U-87 MG and RR cells transfected with mCherry-GFP-LC3B were treated with IR (3 Gy), lidoflazine (1 μM), or both for 24 h. The mean numbers of autophagosomes and autolysosomes are represented by yellow and red dots, respectively, in the merged images. The images were quantified using ImageJ (*n* = 5). Scale bars, 10 µm. **d** Percentage of GBM cells stained positively for the Ki67 proliferation marker undergoing different treatments. **e** Cell reproductive death after treatment with ionizing radiation (3 Gy), lidoflazine (1 µM), or both was determined using crystal violet staining. **f** IC_50_ of lidoflazine with or without IR in U-87 MG RR cells was measured by CellTiter-Glo® Luminescent Cell Viability Assay. Cells were treated with increasing concentrations of lidoflazine and/or IR (3 Gy) for 48 h. **g** Transwell migration and invasion assays were performed to analyze cell movement. Scale bars, 200 µm. **h** Control and RR cells in a three-dimensional culture system were stained with α-tubulin (green) and DAPI (blue). Scale bars, 100 µm. *p < 0.05, **p < 0.01, ***p < 0.001, ****p < 0.0001 using an unpaired *t test*.

To verify the therapeutic effect of lidoflazine in RR cells, the mRNA and protein levels of NRBF2 were measured after treatment with lidoflazine, and both exhibited increased levels in RR cells (Fig. 6a, b). Since an increase in NRBF2 expression causes activation of autophagy, we determined whether the same results were obtained in RR cells. RR cells exhibited high autophagic flux in proportion to NRBF2 levels. In the group treated with lidoflazine after irradiation, the autophagic flux decreased compared to that of the IR group in RR cells (Fig. 6c). In addition, the association between proliferation rate and lidoflazine was examined. Treatment with lidoflazine combined with IR resulted in fewer Ki-67-positive cells and low colony formation (Fig. 6d, e and Supplementary Fig. 4e). We next tested the effect of IR on lidoflazine sensitivity using the CellTiter-Glo® Luminescent Cell Viability Assay (Fig. 6f). IR significantly improved lidoflazine sensitivity in U-87 MG RR cells, reducing the IC50 of lidoflazine from 8.652 μM to 1.983 μM. The effect of lidoflazine on reducing cancer cell aggressiveness, which was demonstrated in U-87 MG cells, was also confirmed in RR cells via a transwell assay, and the same results were obtained in 3D culture alpha-tubulin staining (Fig. 6g, h). Moreover, the role of lidoflazine as a radiosensitizer was demonstrated in RR cells based on our data. Consequently, it was established that the regulation of NRBF2 in radiation therapy is important for increasing sensitivity.

### Lidoflazine modulates GBM growth and survival of mice

**Fig. 7:**
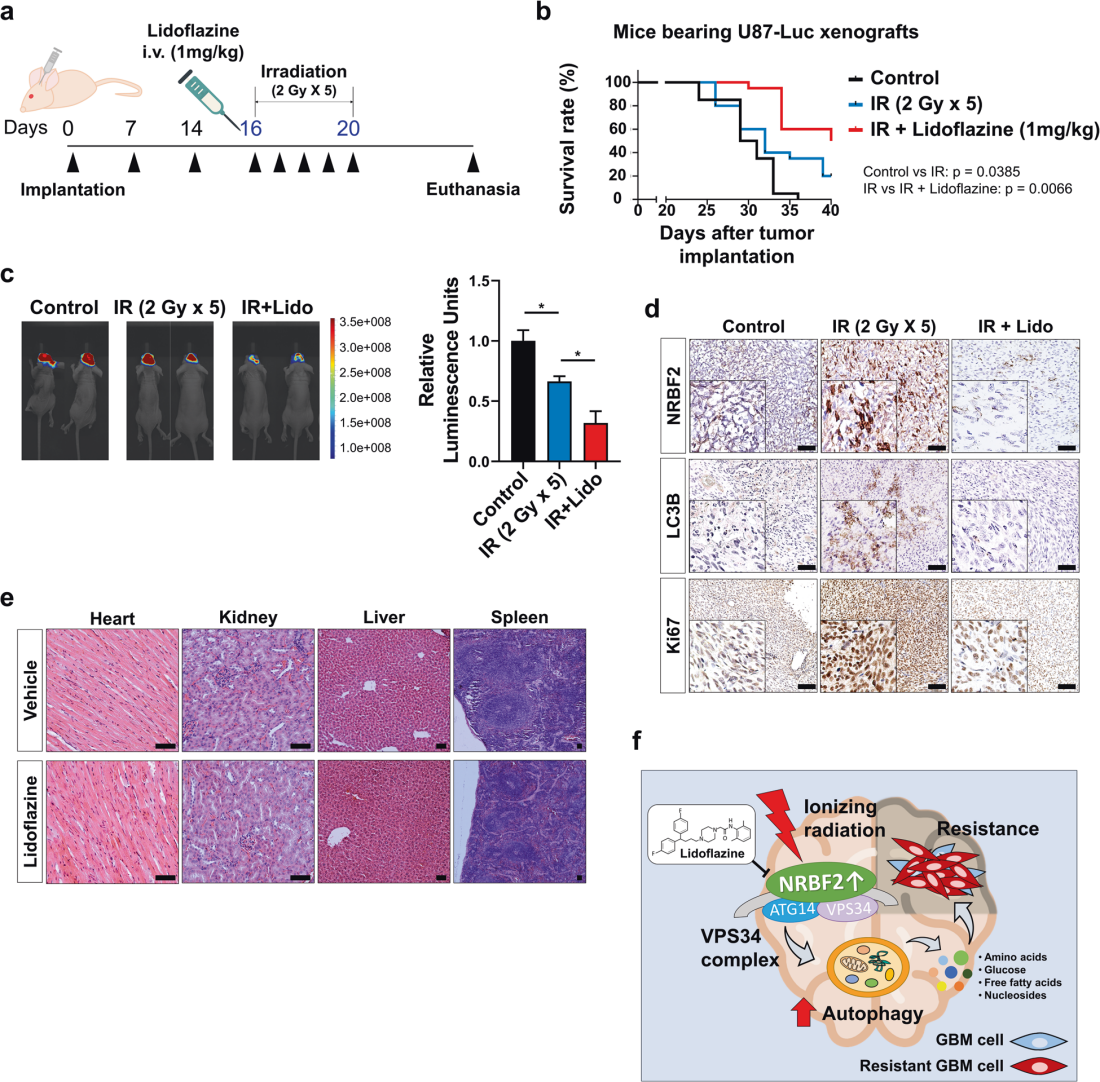
Lidoflazine increases the radiosensitivity of GBM orthotopic xenograft mice. **a** Scheme of the U87-Luc orthotopic xenograft mouse model and mouse treatment schedule. **b** Survival graph of the U87-Luc orthotopic xenograft mouse model with radiation (2 Gy x 5) and lidoflazine (1 mg/kg). Statistical analysis was performed with the log-rank (Mantel‒Cox) test. **c** Luminescence images (left) and relative luminescence units (right) of the indicated U87-Luc orthotopic xenografts after radiotherapy, lidoflazine treatment or not. **d** IHC of NRBF2 (upper), LC3B (middle), and Ki67 (lower) in coronal sections from U87-Luc orthotopic xenograft mouse control or treated with IR (2 Gy x 5) and lidofolfazine (1 mg/kg). Scale bars, 50 μm. **e** H&E staining of organ sections from mice treated with vehicle or lidofructose (1 mg/kg). Scale bars, 50 μm. **f** Schematic diagram illustrating how increased NRBF2 expression upon irradiation leads to GBM resistance.

An orthotopic xenograft mouse model was used to verify the effects of lidoflazine in vitro. Lidoflazine was injected at a dose of 1 mg/kg following radiation treatment (Fig. 7a). Upon examination of the survival rate, the group treated with IR and lidoflazine demonstrated a high survival rate, and the tumor size confirmed via in vivo imaging was also small (Fig. 7b, c). To determine whether this increase in survival rate and decrease in tumor size were due to NRBF2-mediated autophagy, the levels of NRBF2 and LC3B were assessed via IHC analysis. As expected, the levels of NRBF2, LC3B, and Ki67 were increased in the IR (2 Gy × 5 days) group, whereas they were significantly decreased in the combination treatment group (Fig. 7d). Since lidoflazine is a drug that was discovered through drug repositioning, its stability has been verified more thoroughly compared to other newer drug candidates. However, it was found to function as a radiosensitizer rather than as a drug with a mechanism of action. Therefore, confirmation of the side effects on other organs was needed. Histopathological analysis was performed by microscopic observation of each organ tissue (heart, kidney, liver, and spleen), and no cytotoxicity was observed (Fig. 7e). In summary, lidoflazine proved effective in an orthotopic xenograft mouse model and exhibited no toxicity, suggesting a potential novel therapeutic strategy.

## Discussion

GBM is primarily treated with a combination of temozolomide and radiotherapy, which is plagued by poor treatment efficiency and low survival rates. Malignant cancers, including GBM, have been reported to develop resistance to radiotherapy, resulting in decreased efficacy of recent therapies. Therefore, the development of a curative therapeutic approach is necessary. This study observed a tendency for NRBF2 upregulation in response to IR and revealed that NRBF2 causes radioresistance and aggressiveness. An increase in NRBF2 levels activates autophagy, which leads to radioresistance since autophagy supplies the nutrients required by cancer cells. Moreover, it was proven that ATP production and OCR were decreased following irradiation in NRBF2 knockout GBM cells compared to NRBF2 WT GBM cells. This finding indicates that NRBF2-mediated autophagy is related to TCA cycle metabolites and involves oxidative phosphorylation.

Metabolic reprogramming is a hallmark of cancer progression and aggressiveness, wherein cancer cells manipulate their metabolic profile to meet the dynamic energetic requirements of the tumor microenvironment^45,46^. Both palmitate and oleate have been reported to inhibit cell migration^47^, while folate restriction causes metabolic reprogramming and leads to less aggressive cancer^48^. Furthermore, mitochondrial metabolism plays an important role in promoting cell migration and altering cell adhesion, with implications for cancer metastasis^49^. Importantly, a recent study revealed the role of autophagy in epithelial-mesenchymal transition (EMT)-like processes in GBM^50^. Although the role of autophagy in EMT may depend on the cellular context considering its dual role in cancer, this could be strong evidence that altered metabolism by NRBF2 may affect migration and invasion, contributing to autophagy-mediated cancer aggressiveness.

It is necessary to better understand the molecular mechanisms underlying radioresistance in GBM. To this end, we selected NRBF2, which induces autophagy via the VPS34 complex. We found that NRBF2-mediated autophagy is essential for regulating radioresistance in the present study. Nuclear receptor binding factor 1 (NRBF1/MECR), which interacts with peroxisome proliferator-activated receptor alpha (PPARα) and nuclear hormone receptors, is another member of the NRBF family and is a widely known mitochondrial trans-2-enoyl-CoA reductase. Genetic mutations in NRBF1/MECR cause a neurometabolic disorder called MEPAN syndrome in humans^51^. NRBF1/MECR is an enzyme, unlike NRBF2, and this study observed no difference in NRBF1/MECR expression (0.968) in the microarray data. In addition, NRBF1/MECR was not related to autophagy signaling when autophagy regulation genes were identified. This may be because NRBF1/MECR and NRBF2 share only 21.55% identity and 35.13% similarity in their structures^52,53^. NRBF1/MECRit was initially identified as a nuclear receptor binding factor, but as research progressed, it was revealed that another major function was that of an enzyme. Therefore, NRBF2 induced malignancy and radioresistance of GBM through autophagy, which it regulates via its varied functions. NRBF2 is a valuable, novel, and specific treatment target for GBM.

Autophagy is considered a cytoprotective function in cancer therapy and presents a therapeutic resistance mechanism, which is a clinical obstacle to successful cancer treatment while also results in poor prognosis in patients with cancer. Therefore, targeting autophagic pathways has emerged as a potential strategy for cancer therapy. Based on these studies, the combinatorial use of anticancer drugs and hydroxychloroquine (HCQ) has been tested in phase 1 or 2 clinical trial studies, but a clinical trial related to GBM is currently not underway. One GBM study reported that CD133^+^ GSCs induced autophagy after irradiation, and these cells could be radiosensitized by inhibiting autophagy. The use of bafilomycin A1 or shRNA-mediated knockdown of ATG-5 and Beclin-1 resulted in significant radiosensitization of the CD133^+^ population^20^. However, this finding has also been confirmed. Subsequent studies have suggested that transient exposure of GSCs to rapamycin induces cell differentiation and activates autophagy, thereby significantly increasing radiation sensitivity^54^. There are currently no clinical trials on autophagy inhibitors for the control of radioresistance, but it has also been reported that autophagy induces radioresistance in several carcinomas, along with drug resistance^55,56^. This proves that a more in-depth study of the relationship between radioresistance and autophagy is needed. It also suggests that it is necessary to discover and control specific factors or signals that cause radioresistance induced by autophagy rather than simply inhibiting autophagy.

Drug discovery is time- and cost-intensive; hence, drug repositioning has increased in popularity in recent years. Lidoflazine, originally used as a vasodilating agent, is a calcium channel blocker. This drug can prolong the QT interval and attenuate calcium accumulation in neurons after ischemia (ISBN 9780323073073, ISBN 9780444537164). This study utilized lidoflazine as a radiosensitizer for the treatment of GBM. Radiosensitizers are drugs or chemical compounds, including chemotherapy agents that boost the effects of radiotherapy^57^. They comprise several types of molecules, such as proteins, peptides, miRNAs, and siRNAs, broadly called macromolecule radiosensitizers or nanomaterials. The effectiveness of lidoflazine after irradiation was verified via a clonogenic assay, Ki67 and cytoskeleton staining, western blotting, qRT‒PCR, transwell assay, and autophagic flux assays. Cells that received isolated treatment with this drug exhibited no difference, but combination therapy with IR highly reduced cancer cell proliferation and aggressiveness, as depicted in Figs. 5 and 6. These results were obtained from the NRBF2 reduction observed when the mRNA and protein levels of NRBF2 were evaluated. Consequently, it was found that autophagic flux was low because of a decrease in NRBF2 expression. These findings suggest that lidoflazine can reduce NRBF2-mediated autophagic flux, which accelerates radioresistance during radiation therapy and enhances therapeutic efficacy. Additionally, it was expected that lidoflazine would be effective when treatment with lidoflazine alone was administered to RR cells; however, it showed the greatest efficacy following combinatorial treatment with IR. Thus, it presumably acts as a sensitizer when combined with IR, and it can be concluded that NRBF2 causes radioresistance.

Autophagy provides metabolic substrates for both biosynthesis and energy generation^58^. In terms of energy generation, oxidative phosphorylation is considered to be more important than glycolysis because amino acids and lipid species generated by autophagy can only be metabolized by oxidative phosphorylation, and it is a more efficient metabolic process for ATP production^58,59^. Accordingly, our metabolic analysis showed that IR-induced NRBF2 increases oxidative phosphorylation but has little effect on the glycolytic rate. Furthermore, TCA cycle intermediates and ATP levels were highly downregulated by NRBF2 knockdown. Therefore, our data demonstrate that NRBF2-mediated autophagy activates oxidative phosphorylation by providing TCA cycle intermediates to acquire radioresistance in GBM. Moreover, to enhance therapeutic effectiveness, lidoflazine was selected, and its inhibitory effect on GBM aggressiveness and proliferation was confirmed. The present study provides new insights that prove beneficial for regulating the adverse effects of GBM radiotherapy and elevating its efficacy against radioresistance (Fig. 7f).

## Supplementary information

Supporting Information
