# Supplementary material for: NRBF2-mediated autophagy contributes to metabolite replenishment and radioresistance in glioblastoma
Source: Exp Mol Med. 2022 Nov 4;54(11):1872–85. doi: 10.1038/s12276-022-00873-2 (PMC9723115; doi:10.1038/s12276-022-00873-2)
Supplement: Supplementary file 1 — Supporting Information [file 12276_2022_873_MOESM1_ESM.pdf]

Supporting Information (SI)

Supplementary figure

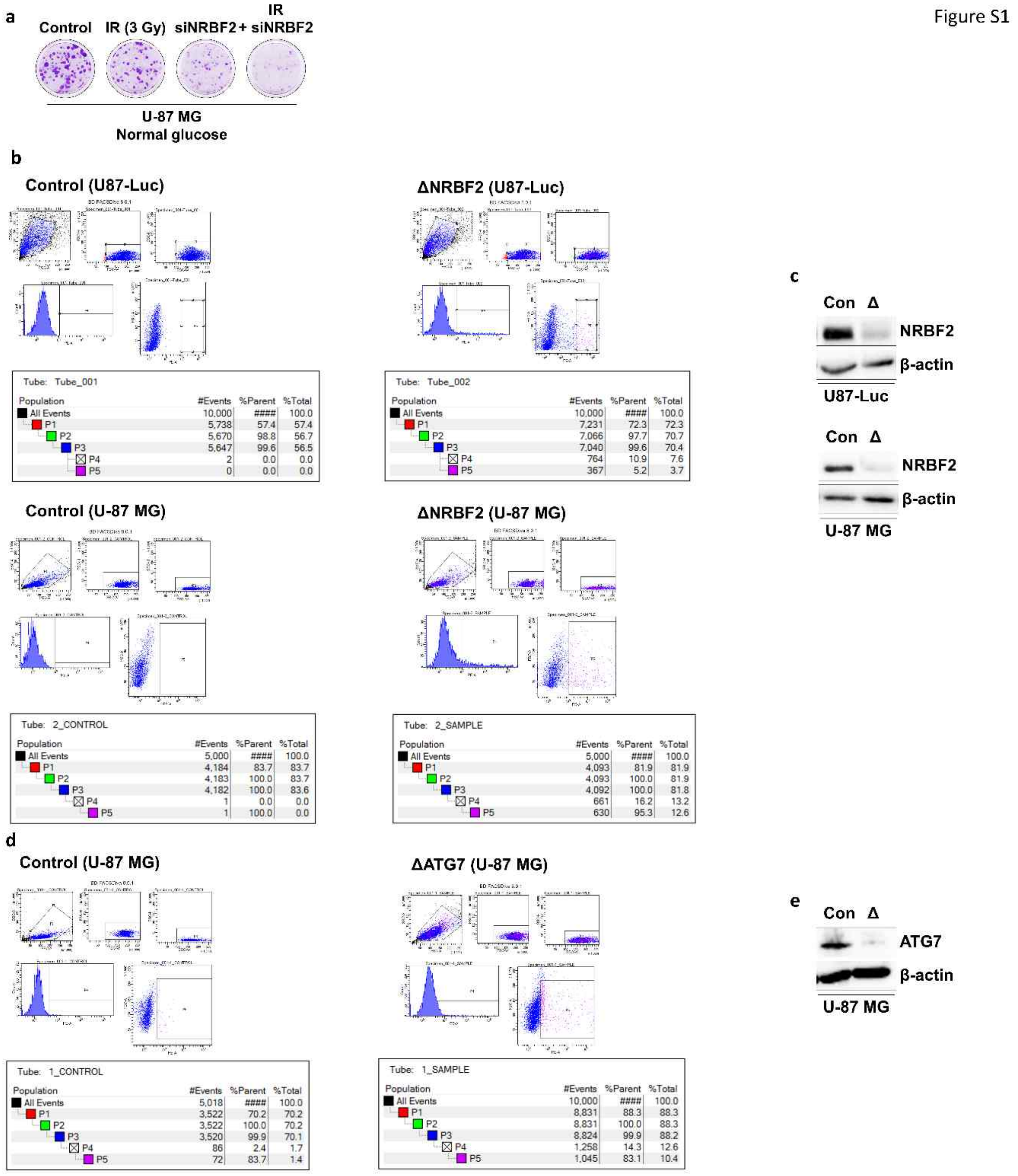

Supplementary Fig 1. **a.** Clonogenic assay after NRBF2 knockdown or IR (3 Gy) or both in normal glucose condition. **b-e.** NRBF2 (b) or ATG7 (d) knockout cell isolation through FACS and protein level of NRBF2 (c) or ATG7 (e) confirmed by western blot.

Figure S2

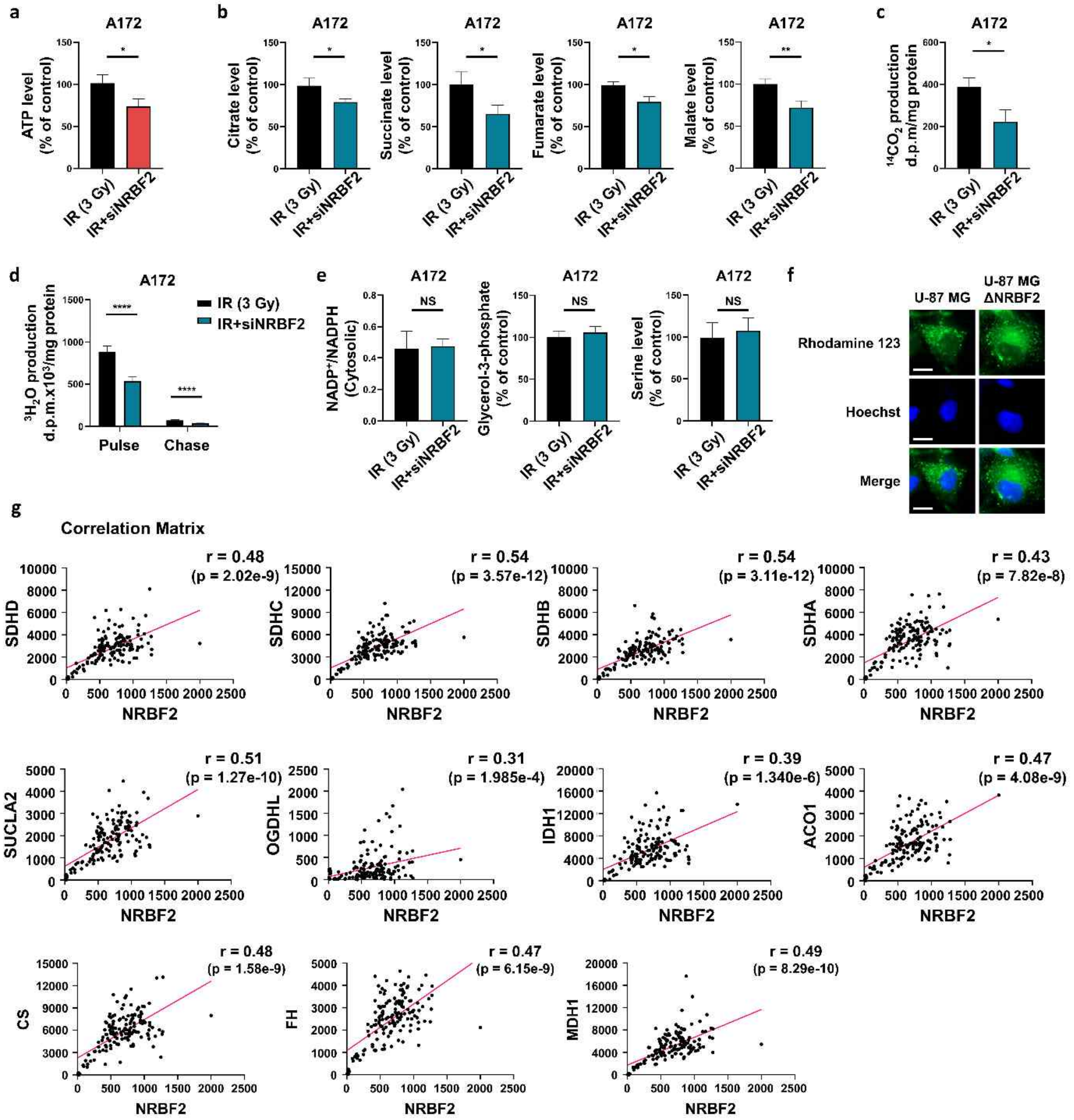

Supplementary Fig 2. a-e. Each bar graph manifests metabolite levels between WT and NRBF2 knockdown A172. (a) ATP levels were determined after irradiation (3 Gy). (b) TCA cycle metabolites such as citrate, succinate, fumarate, and malate were detected. (c) WT and NRBF2 knockdown A172 cells were incubated with [1-^{14}C] oleic acid and the $^{14}CO_2$ production from the complete b-oxidation of [1-^{14}C] oleic acid was analyzed. (d) After incubation with [9,10-^{3}H] oleic acid (pulse) followed by incubation in RPMI (chase), media equivalent to 500ug of cell protein were analyzed for radioactivity in the form of ^3H_2O. (e) Metabolites of glycolysis-related pathways were measured by measuring NADP$^+$/NADPH ratio, glycerol-3-phosphate, and serine levels. **f**. Representative images of mitochondrial membrane potential in U-87 MG and U-87 MG ΔNRBF2 through Rhodamine 123 staining. Scale bars, 10 μm. **g**. The gene correlation profiles between NRBF2 and TCA cycle enzymes were collected from The Cancer Genome Atlas (TCGA) database available through cBioPortal (http://cbioportal. org). *$p < 0.05$, **$p < 0.01$, ****$p < 0.0001$ using unpaired t-test.

Figure S3

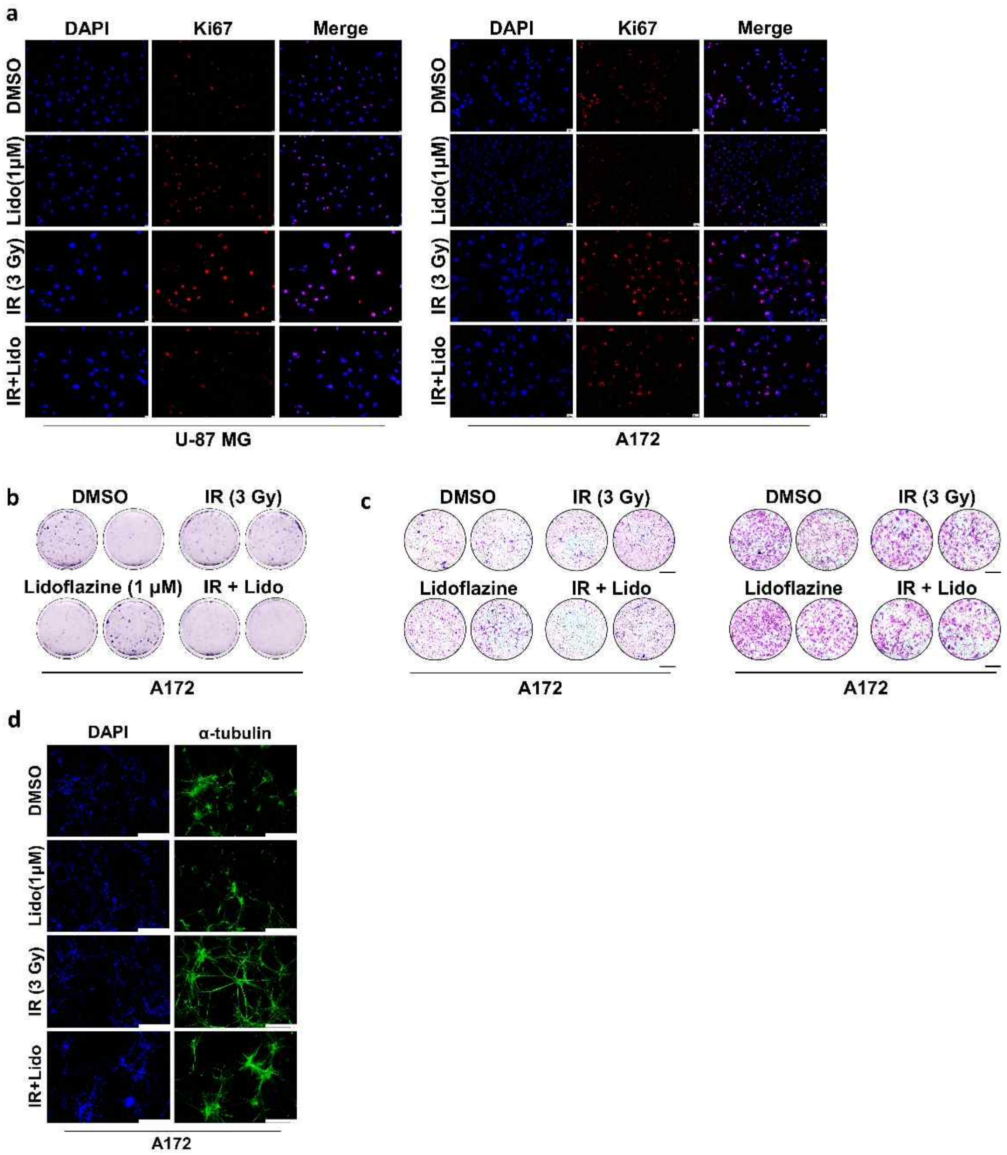

Supplementary Fig 3. a. Immunofluorescence microscopy of Ki-67-stained U-87 MG and A172. Bright red staining visualized in the images indicates positive staining for Ki-67, while blue represents counterstaining of cell nuclei with DAPI. Ki67-positive cells were counted and represented by a bar graph. Scale bars, 10 μm. **b.** Clonogenic assay after IR (3 Gy), lidoflazine (1μM), or both in A172 cells. **c.** Transwell migration assay (left) and invasion assay (right) of A172 cells with treatment lidoflazine (1μM), IR (3 Gy), or both. **d.** A172 cells in a three-dimensional culture system were stained with α-tubulin (green) and DAPI (blue). Scale bars, 50 μm.

Figure S4

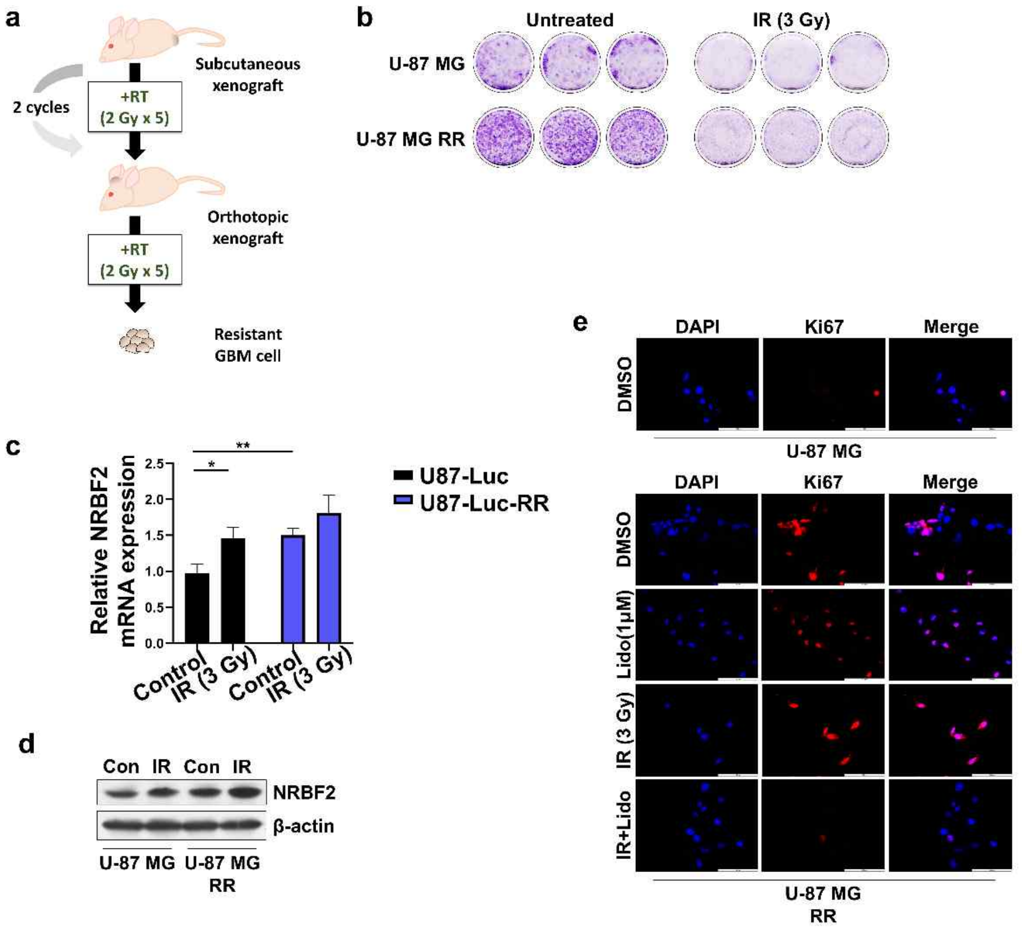

Supplementary Fig 4. a. Scheme of radioresistant cell establishment. **b**. Clonogenic assay after untreated (left) or IR (3 Gy, right) in U-87 MG and radioresistant (RR) cells. Representative images of single-cell clone proliferation stained with crystal violet. **c**. qRT-PCR analysis of *nrbf2* mRNA levels after irradiation in control and RR cells. **d**. Western blot analysis to check NRBF2 protein expression level after IR (3 Gy). **e.** Immunofluorescence microscopy of Ki-67-stained U-87 MG and A172. Bright red staining visualized in the images indicates positive staining for Ki-67, while blue represents counterstaining of cell nuclei with DAPI. Ki67-positive cells were counted and represented by a bar graph. Scale bars, 100 μm.

Supplementary Table 1. Patient information of patient-derived GBM cell line

Cell line	BCL20-HP01	BCL20-HP02	BCL21-HP03
Sex	M	M	M
Age (years)	47	38	67
Pathology	Glioblastoma	Glioblastoma	Glioblastoma
IDH1	Wild-type	Wild-type	Wild-type
MGMT	Positive	Positive	Positive
Neu-N	Negative	Negative	Negative
GFAP	Diffuse positive	Diffuse positive	Strong positive
Oligo-2	Focal positive	Positive for tumor cell nuclei	Focal positive
p53	1.4% positive	10% positive	87.2% positive
Ki-67	18.9% positive	29.3% positive	68.8% positive

Supplementary Table 2. Primer sequence of qRT-PCR

Gene	Sequence
NRBF2 Forward	5′- CTTGGCAGACGCTAAACTCATG -3′
NRBF2 Reverse	5′- CCAGGTAGCCATTTGGTTTGTAT -3′
GAPDH Forward	5′- ATGACATCAAGAAGGTGGTG -3′
GAPDH Reverse	5′- CATACCAGGAAATGAGCTTG -3′
